# Gaussian Model of Anti-Radar Properties for Coatings Based on Carbonyl Iron Powder

**DOI:** 10.3390/ma16083050

**Published:** 2023-04-12

**Authors:** Wojciech Przybył, Robert Mazurczuk, Artur Kalinowski, Krzysztof A. Bogdanowicz

**Affiliations:** 1 Military Institute of Engineer Technology, Obornicka 136, 50-961 Wrocław, Poland; 2 Faculty of Mechatronics and Mechanical Engineering, Kielce University of Technology, Al. 1000-lecia P. P. 7, 25-314 Kielce, Poland

**Keywords:** radar absorption, carbonyl iron coatings, military application, epoxy resin

## Abstract

The article presents the Gaussian model of the electromagnetic radiation attenuation properties of two resin systems containing 75% or 80% of a carbonyl iron load as an absorber in the 4–18 GHz range. For the attenuation values obtained in the laboratory, mathematical fitting was performed in the range of 4–40 GHz to visualize the full curve characteristics. The simulated curves fitted up to a 0.998 R^2^ value of the experimental results. The in depth analysis of the simulated spectra allowed a thorough evaluation of the influence of the type of resin, absorber load, and layer thickness on reflection loss parameters such as the maximum attenuation, peak position, half-height width, and base slope of the peak. The simulated results were convergent with the literature findings, allowing a much deeper analysis. This confirmed that the suggested Gaussian model could provide additional information, useful in terms of comparative analyses of datasets.

## 1. Introduction

**Figure 1:**
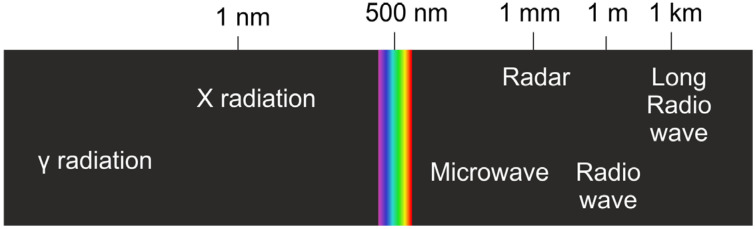
Scheme of the spectrum of electromagnetic radiation.

Today’s armed conflicts, especially full-scale ones, confirm that the use of armored weapons, gun and rocket artillery, and aviation is essential to achieve operational and tactical goals. The basis for the effectiveness of these types of weapons is effective reconnaissance [1]. Thanks to the widespread use of various sensors, recognition is carried out with a wide range of electromagnetic radiation (Figure 1).

Optical and thermal bands are usually used for closer ranges, whilst radar bands are commonly used for longer ranges. The radar range covers wavelengths from a few millimeters to several meters and is divided into a number of bands, depending on the propagation properties (Table 1).

Several bands are used in applications related to military reconnaissance; mainly, C (4–8 GHz), X (8–12 GHz), and Ku (12–18 GHz). Thus, the authors focused on the radar properties of materials for these frequency bands. The aim of the authors was to build a mathematical model that, for a material with radio wave-damping properties for the above-mentioned ranges, would make it possible to determine the frequency for which the maximum attenuation would occur and its level, depending on the parameters of the paint coating; i.e., the thickness, percentage share, and type of binder. A number of radiation absorbers are now known. These are metals (nickel, iron–nickel alloys, and iron), carbon-based non-metals (graphite, graphene, and their nanotubes, nanoparticles, nanofibers, and flakes) [3,4,5,6,7], ceramic materials (magnetite), conductive polymers (PEDOT:PSS [8]), and composites.

The total attenuation of materials can be described by the shielding efficiency SE (dB), which is described by the following equation [9]:

*SE* = 10 log|*P_I_*/*P_T_*| = 20 log|*E_I_*/*E_T_*| = 10 log|*H_I_*/*H_T_*| (1)

where *P_I_* and *P_T_* are the incident power (*I*) and emitted power (*T*), respectively; *E_I_* and *E_T_* are the intensity of the incident (*I*) and emitted (*T*) electric field, respectively; and *H_I_* and *H_T_* are the intensity of the incident (*I*) and emitted (*T*) magnetic fields, respectively.

Shielding effectiveness can be broken down into a factor of reflection, absorption, and multiple internal reflections:

*SE* = *SE_R_* + *SE_A_* + *SE_MR_* (2)

where *SE_R_* is the factor caused by reflection (dB), *SE_A_* is the factor caused by absorption (dB), and *SE_MR_* is the factor caused by multiple reflections.

The factor due to reflection can be described by the following equation [10]:

*SER* = −10 log(*σT*/16*ωε*_0_*μ_r_*) (3)

where *σ_T_* is the total material conductivity, *μ_r_* is the relative magnetic permeability, *ω* is the frequency, and *ε*_0_ is the electric permeability of the vacuum.

The factor caused by the absorption is described by the following equation [10]:

*SE_A_* = −8.68*t*(*σ_T_ωμ_T_*/2)^1/2^ (4)

where *σ_T_* is the total material conductivity, *μ_r_* is the relative magnetic permeability, and *ω* is the frequency.

On the other hand, the factor caused by internal reflection for shells larger than the so-called skin depth can be described by the following equation [10]:

*SE_MR_* = 20 log|(1 − 10^−*SE_A_*/10^)| (5)

The last equation shows that, for materials with good absorbing properties, the factor caused by internal reflection from external surfaces can be neglected, which is the case in practice.

Carbonyl iron with spherical particle sizes of 3–4 μm was used in this work. This was suspended in epoxy resin, forming a paste to be applied as a varnish coating for painting masking military equipment.

In the literature on the subject [11,12,13], one can find reports detailing that the microwave permeability for systems built of dispersed ferromagnetic material (carbonyl iron) in a dielectric (epoxy resin) also depends on the size and proportion of the ferromagnetic particles and may be periodic in the frequency domain, which may result, for example, in the mutual interference of the absorber particles suspended in the binder.

Thus, modelling the attenuation values for wide frequency ranges and for two and more phase systems is quite complicated. In this paper, the authors used the radar equation and attempted to determine the relationship for the frequencies most often used in military reconnaissance; i.e., 4–18 GHz.

(6) $$P_{RX}=\frac{P_{TX}\cdot G_{TX}\cdot G_{RX}\cdot \sigma \cdot \lambda^{2}}{{\left(4\pi \right)}^{3}R^{4}}$$

where *G_RX_* is the gain of the receiving antenna, *G_TX_* is the gain of the transmitting antenna, *P_RX_* is the signal strength of the receiving antenna, *P_TX_* is the signal strength of the transmitting antenna, *λ* is the wavelength, *σ* is the effective reflection area, and *R* is the distance of the object from the antenna (assuming that we are dealing with a monostatic system, where the transmitting and receiving antennas are at the same distance).

Using Equation (1) and transforming Equation (6) and treating the sample as a wave emitter, we obtained:

(7) $$\mathrm{SE}=10\;\log|P_{I}/P_{T}|=SE=10\;log\left|\frac{P_{I}}{P_{T}}\right|=10\log\left|\frac{{\left(4\pi \right)}^{3}R^{4}{}_{}}{G_{TX}\cdot G_{RX}\cdot \sigma \cdot \lambda^{2}}\right|$$

Assuming that the tests would be carried out in the same conditions, for samples of the same size, Equation (7) may take the form:

(8) $$SE=10\;\log|k/(\sigma \cdot \lambda^{2})|,$$

where *k* is a constant and *σ* depends on the size and geometry of the object, type of material, layer thickness, and wavelength *λ*. For constant sizes of the tested object and the standard one, the factor related to the geometry of the object was constant. Thus, by measuring the *SE* for known wavelengths *λ* and the thickness of the layer, one can obtain a model of dependence of the attenuation of the frequency *f*, the thickness *d* of the absorber layer, and the share of the absorber:

*σ*(*f, d, λ*) = f(*SE*, 10 log(*λ*^2^)) (9)

In this study, we analyzed a set of four anti-radar paint compositions based on two different resins, a chemical hardener, an improver, and two absorber contents. The paints were used for thin coatings. For each composition, four different layers with thicknesses of 0.5 mm, 1.0 mm, 1.5 mm, and 2.0 mm were made on an aluminum sheet with dimensions of 300 mm × 300 mm × 3 mm. The aims of this article were to propose, for the first time, a Gaussian model to characterize the anti-radar properties, and to perform an evaluation of mathematical model in terms of the curve factors (signal width, maximum attenuation, and frequency of the maximum attenuation) in relation to the layer composition and layer thickness. The aim of modeling was also to determine the influence and trends of the physical parameters of paints on their attenuation in the microwave range of 4–40 GHz, based on experimental values obtained for the range of 4–18 GHz.

## 2. Materials and Methods

Sets of pastes were prepared, differing in the content of the absorber (carbonyl iron, purchased from BASF Aktiengesellschaft, and Carbonyleisenpulver EB, marked as EB) and the binder used (epoxy resin with a chemical hardener and, if necessary, an improver, viscosity-reducing agent, and plasticizer), as shown in Table 2. It is worth noting that the sizes of the absorber particles (EB) was 3–4 μm.

For each composition, four different layers with thicknesses of 0.5 mm, 1.0 mm, 1.5 mm, and 2.0 mm were made on an aluminum sheet with dimensions of 300 mm × 300 mm × 3 mm.

**Figure 2:**
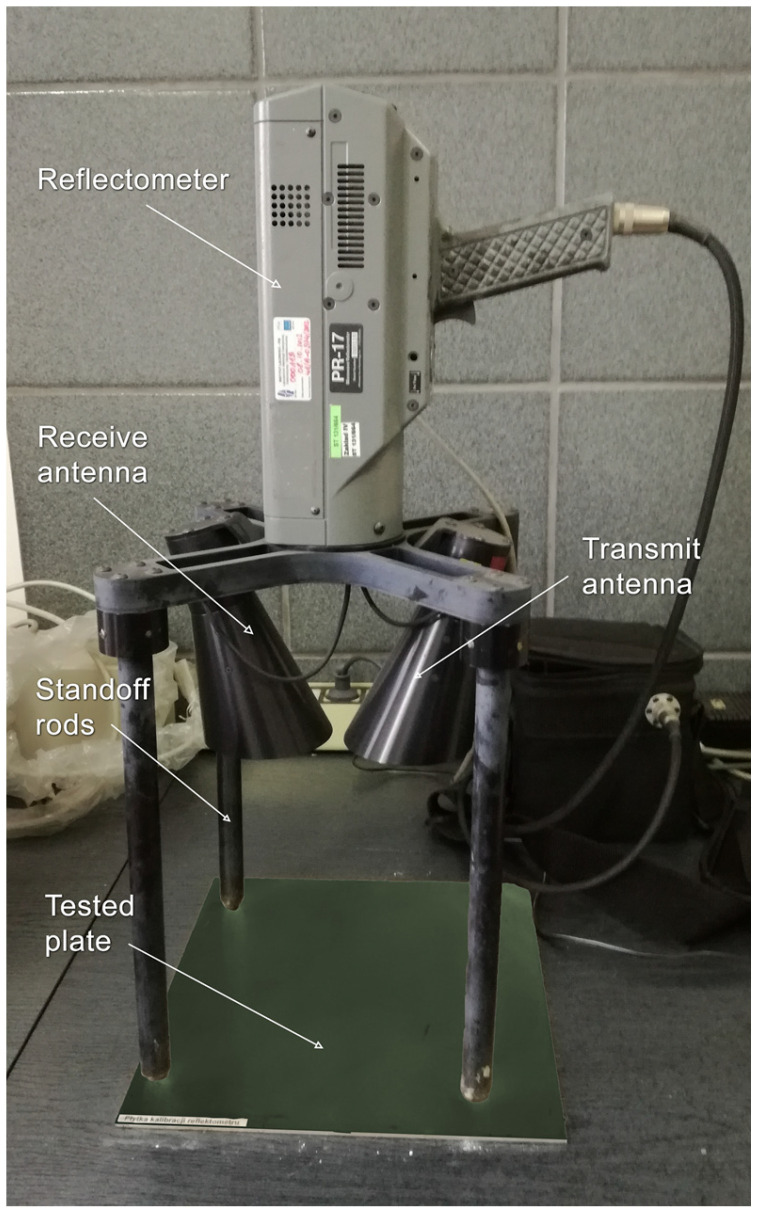
Scheme of the testing setup.

The attenuation tests were carried out on a measuring stand using a PR-17 CXKU reflectometer (Milimeter Wave Technology INC, Marietta, GA, USA) operating in a range from 4 to 18 GHz (Figure 2). 

The result of the tests was the attenuation level of the samples expressed in dB and related to the reference aluminum plate without any coating. Details of the research are presented in Table 3 and [14].

## 3. Results and Discussion

All tested samples containing the absorber showed attenuation characteristics with one dominant maximum and were similar to the shape of the Gaussian curve.

In order to simulate the attenuation characteristics of electromagnetic radiation in the microwave range, the following assumptions were made:

1. A frequency range from 4 GHz to 40 GHz was adopted for the model attenuation calculations;
2. Based on the experimental data, one dominant damping maximum was assumed for the simulated model;
3. A damping model with Gaussian curve characteristics was adopted for each sample;
4. A simulation was made for the maximum attenuation, preceded by the minimum recorded in the range from 4 to 18 GHz;
5. Due to the location of the measurement points with maximum attenuation near the limits of the 4 GHz and 18 GHz measurement ranges for several samples, an additional approximation correction was introduced. The first point with the maximum attenuation level after the minimum was considered to be the maximum for which each subsequent point assumed an attenuation value equal to the maximum value with a tolerance of ±0.3 dB.

A script created in the Python programming language was used to calculate the mathematical model. The *leastsq* function from the optimize programming library belonging to the SciPy module was used. The *leastsq* function is an iterative function using Jacobians based on the difference between the observed objective data (measurements for *x*) and a defined non-linear function of the parameters *f*(*x*, *coefficients*), where the least squares approach is used to minimize [15].

The mathematical model was used to calculate the formula of the Gaussian curve function described by Formula (10):

(10) $$f\left(x\right)=B+A\;exp\left[-{\left(\frac{x-\mu }{\delta }\right)}^{2}\right]$$

where *x* is the variable frequency value; the equation coefficient *B* is the noise value, the baseline defining the minimum attenuation value; *A* is the amplitude, the highest attenuation value measured from the baseline; *δ* is the conventional peak width, satisfying the relation to a half-width FWHM ≈ 2.35 *δ*; and *μ* is the peak midpoint, the frequency for the maximum attenuation value.

In order to compare and normalize the indication of one universal best absorbing composition determined by the share of the absorber, the thickness of the coating, and the type of binder in the frequency range of 4–18 GHz, a statistical analysis of samples meeting the following exemplary conditions was also performed:

1. Attenuation level exceeding 12 dB;
2. Range with maximum attenuation in the range of 8–12 GHz (X band).

### Mathematical Simulation

Based on the experimental data for samples of various thicknesses made with paints consisting of two binder systems based on different epoxy resins and containing 75% and 80% carbonyl iron as an absorber, all tested samples showed damping characteristics with one dominant maximum and taking the shape of a Gaussian curve.

It was also noticed that the curves in the graphs had a shape similar to Gaussian curves with different amplitudes, midpoints, and peak widths. Therefore, mathematical simulations were performed to determine the dependence of the attenuation value on the frequency for each measurement sample. This made it possible to obtain the coefficients of the Gaussian curve equation, which had their reference in the physical properties of the tested samples. It was, therefore, assumed that the model Gaussian curve was described by Equation (9).

In the first stage of the simulation, the initial values of the equation coefficients were determined, assuming *B*_0_ = 0, *A*_0_ was the value of the maximum attenuation derived from the measurements for a given sample, *δ*_0_ was the entire width of the tested range, and *μ*_0_ was the frequency for which the maximum attenuation occurred. The implemented Levenberg–Marquardt algorithm using the method of successive approximations, changing the coefficients of the equation and minimizing the value of the mismatch based on the sum of the least squares of difference between the values from the measurements and the model curve, determined the equation coefficients for the model curve.

In accordance with the assumptions, the occurrence of one damping maximum in the considered range was assumed, which was used for further analyses.

Similarly, when the measurement points with maximum attenuation occurred near the limits of the 4 GHz and 18 GHz measurement ranges, an additional approximation correction was introduced, taking as the maximum the first point with the maximum attenuation level following the minimum for which each subsequent point had an attenuation value equal to a maximum value of ±0.3 dB.

In addition, due to the adopted model, the Gaussian curve, and its symmetry, in the case of a clear maximum attenuation within the measurement range, the part of the data that corresponded with a larger number of measurement points was used for modelling.

The results of fitting the model curves to the measurement results along with the correlation coefficients are presented in Table 4.

**Figure 3:**
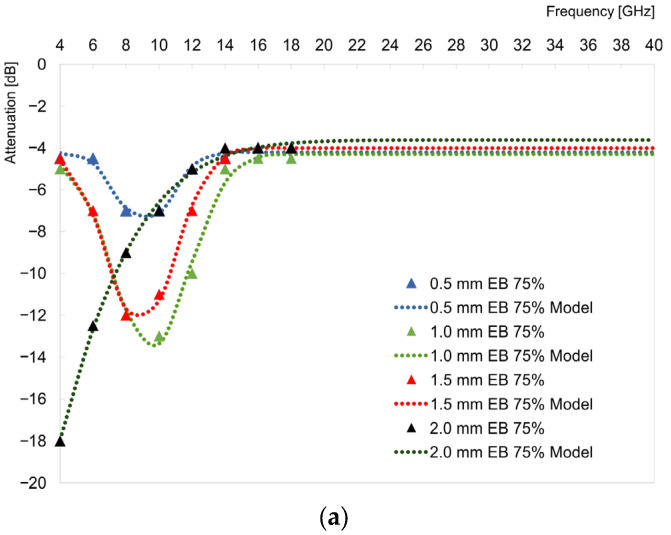

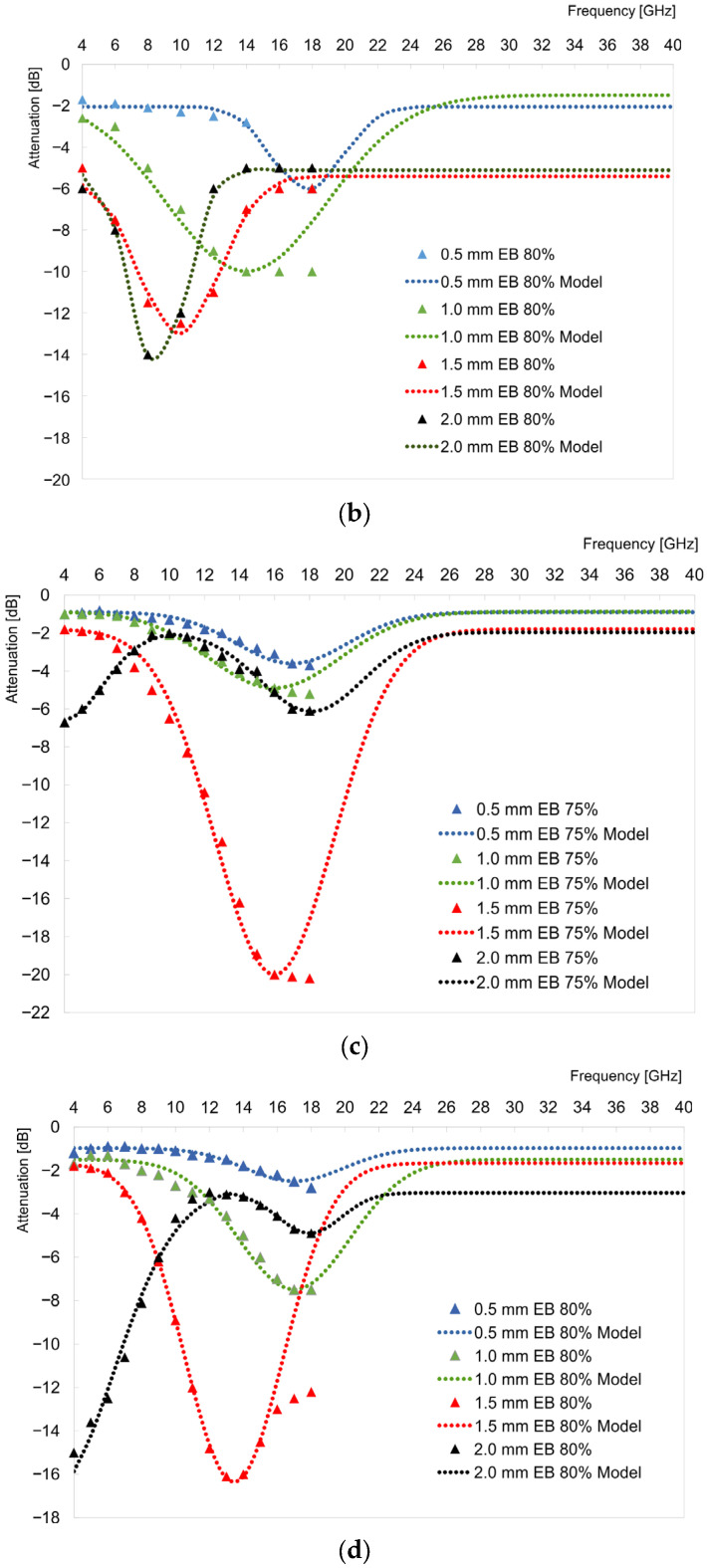
Visualization of results from measurements and modeling for resin L1 and 75% absorber content (**a**), resin L1 and 80% absorber content (**b**), resin L2 and 75% absorber content (**c**), and resin L1 and 80% absorber content (**d**).

The graphs in Figure 3 show the measurement values in the 4–18 GHz range and model curves in the wider range of 4–40 GHz in order to visualize and adjust the Gaussian curve to the measurements whose maxima were within the measurement range. The extended characteristics were beyond the experimental range (Figure 3).

The samples made of both L1 and L2 resins tended to display a broad attenuation signal and only slight changes in the width of the peak depending on the thickness were noticed. All layers showed a similar trend of peak slope change, consisting of an increase in the peak slope value depending on the layer thickness; however, for the layer with the L2 resin, it reached a maximum for a thickness of 1.5 mm and then decreased.

With regard to the noise level, a tendency was observed; in the system with the L1 resin where additional components were included, i.e., plasticizers and viscosity-lowering agents, there was no clear trend in relation to the layer thickness and the corresponding noise level. On the other hand, in the case of the L2 set based on a monomer (resin) and an activating agent (curing agent), there was a relationship between the increase in the noise level and the increase in the thickness of the coating (Figure 3). Additionally, a difference in the shape of the observed curves was noticed; in the case of the 2 mm thick layer and the L2 resin only, two signals were noticed instead of one, similar to the layers below 2 mm thick and the L1 resin systems. This might have been related to the uneven distribution of the absorber in the layer. The system behaved as if it contained an additional layer of a spacer or an absorber of a poorer section. A similar behavior was observed by Yuping et al. [16] for a system containing polyurethane–carbonyl iron particles (PU-CIP)/polyvinyl chloride(PVC)/aluminum. It was observed that there was one sharp and one broad signal, approximately located at frequencies 2–4 GHz and 8–16 GHz, respectively. The authors also noticed that the reflection losses were similar for both variations with a fixed thickness of PVC or PU-CIP.

The layers showed a similar tendency to change the maximum attenuation value, consisting of an increase in the attenuation level with an increase in the layer thickness. This tendency was visible in the case of the layers containing 80% absorber in the coating composition (Figure 3b,c). In the case of 75% absorber content, the tendency was not so clear (Figure 3a,d). In the case of the L1 resin with 75% absorber, the result for the 1.5 mm thickness did not fit the expected trend, showing slightly lower values of attenuation in relation to the 1 mm sample. 

**Figure 4:**
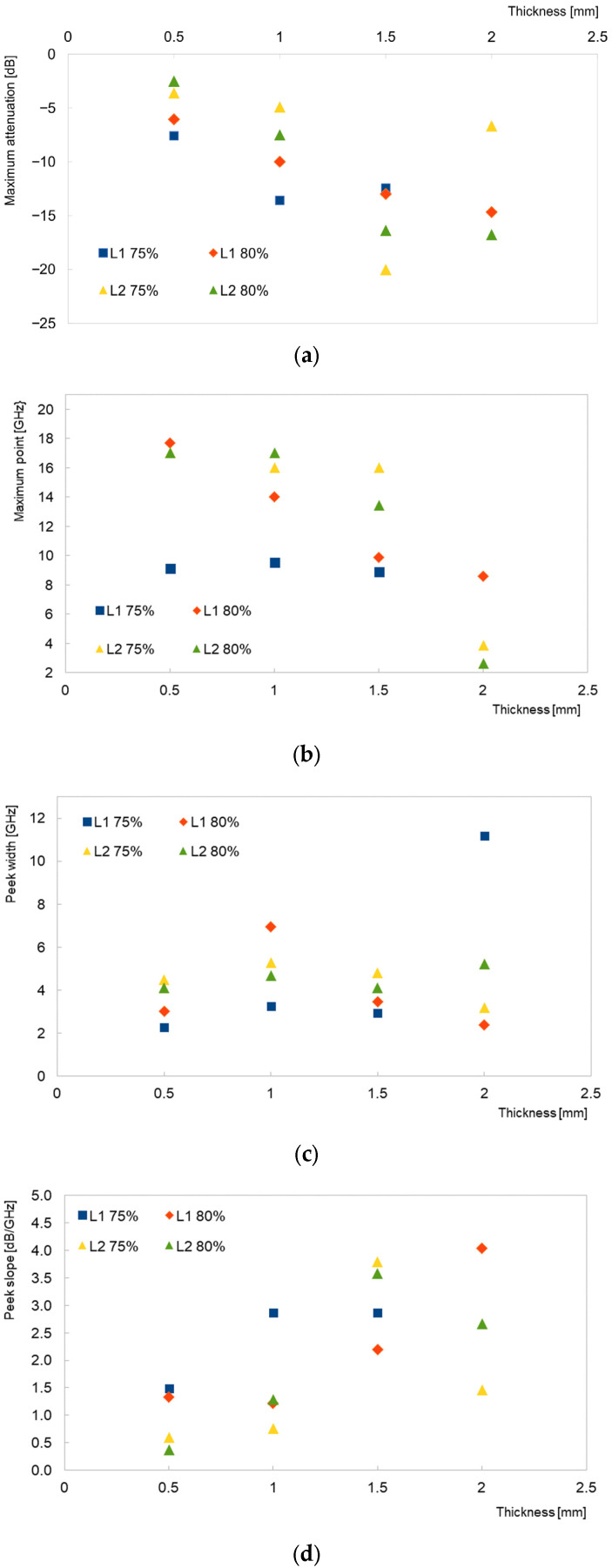
Relationship of layer thickness vs. selected factors: maximum attenuation (**a**); frequency of maximum of attenuation (**b**); signal width (**c**); and curve slope (**d**).

The visualized trends of changes in the coating parameters according to the model curves are shown in Figure 4. As anticipated, the increment in the thickness of the coating resulted in an increment of the attenuation of the initial signal (Figure 4a). From the microwave absorption theory, it is known that when the electromagnetic wave strikes a material, the incident power can be divided into reflection, absorption, and transmission. The reflection occurs on the surface of the conductive layer, generating multiple internal reflections. In the case of absorbing materials, the energy can be absorbed by the absorber or can bypass the external coating, reaching the lower surface and causing multiple internal reflections [17]. Our understanding was that the high content of carbonyl iron as an absorber resulted in a good transmission of the signal into the layer, which was absorbed via conductivity and magnetic dipoles dispersed in the resin matrix, as described in the literature [2]. 

When the position of the maximum was evaluated in the case of the L1 and L2 resins and 75% load only, the signal remained centered around 10 GHz and 16 GHz, respectively, for the layer thicknesses in the range of 0.5 to 1.5 mm. For the 2 mm thick layer, the expected maximal signal was located below 4 GHz and was not simulated. The difference in the layer thicker than 1.5 mm might suggest that for 2 mm layers, the arrangement of the carbonyl iron load in the resin matrix could form a few aggregates; hence, there was a step change in the position of the maximum of the reflection loss.

In Figure 4c, the relationship between the width of the peak at the half-height is present. It could be observed that, for most of the samples, this parameter did not significantly change with the thickness of the layer. The same result was observed in the literature; however, no comments were given on the subject [16].

## 4. Conclusions

In conclusion, the radar-absorbing properties of composite coatings based on a carbonyl iron-coated Al sheet were studied in the 2–18 GHz range. To simulate the reflection losses, a Gaussian mathematical model was employed to simulate the absorption characteristics of the material in wider frequency spectra of up to 40 GHz. The obtained mathematical model fitted very well with the experimental results, reaching R^2^ values between 0.891 and 0.998. The simulated curves allowed a more accurate comparison of the obtained experimental results.

The analysis of the simulated signals confirmed the typical spectral characteristics of reflection losses typical for carbonyl iron observed in the literature. Moreover, the correlation between the thickness of the layer for two resin systems and two different payloads of absorbers showed that the attenuation value increased with the increasing layer thickness, slightly shifting the maximum peak towards longer wavelengths. Furthermore, peak parameters such as the width at the half-height were maintained at the same level, increasing with the level thickness peak slope. 

All the above-mentioned findings confirmed the validity of Gaussian curve modelling for carbonyl iron-based systems with high accuracy.

## Figures and Tables

**Table 1:** Frequency division into bands [2].

Band Name	Frequency (GHz)	Wavelength (cm)	Band Symbol
VHF	0.1–0.3	300–100	A
UHF	0.3–0.5	100–60	B
	0.5–1.0	60–30	C
L	1–2	30–15	D
S	2–3	15–10	E
	3–4	10–7.5	F
C	4–6	7.5–5	G
	6–8	5–3.75	H
X	8–10	3.75–3	I
	10–12	3–2.5	J
Ku	12–18	2.5–1.67	J
K	18–26.5	1.67–1.1	J (to 20 GHz)
Ka	26.5–40	1.1–0.75	K
Millimeter waves	40–100	0.75–0.3	L (to 60 GHz)
			M (>60 GHz)

**Table 2:** List of paint compositions.

No.	Resin	Chemical Hardener	Viscosity-Reducing Agent/ Plasticizer	Absorber Content
1	Shell Epikote 828 (L1)	Ancamine 1618	AX-R/AX-S	75%
2	Shell Epikote 828 (L1)	Ancamine 1618	AX-R/AX-S	80%
3	Epidian 112 (L2)	Saduramid 10/50	—/—	75%
4	Epidian 112 (L2)	Saduramid 10/50	—/—	80%

**Table 3:** Comprehensive list of attenuation values (dB).

Frequency (GHz)	Absorber Content 75%				Absorber Content 80%				Resin
	0.5 mm	1.0 mm	1.5 mm	2.0 mm	0.5 mm	1.0 mm	1.5 mm	2.0 mm	
4	−4.5	−5	−4.5	−18	−1.7	−2.6	−5	−6	L1
6	−4.5	−7	−7	−12.5	−1.9	−3	−7.5	−8	L1
8	−7	−12	−12	−9	−2.1	−5	−11.5	−14	L1
10	−7	−13	−11	−7	−2.3	−7	−12.5	−12	L1
12	−5	−10	−7	−5	−2.5	−9	−11	−6	L1
14	−4.5	−5	−4.5	−4	−2.8	−10	−7	−5	L1
16	−4	−4.5	−4	−4	−5	−10	−6	−5	L1
18	−4	−4.5	−4	−4	−6	−10	−6	−5	L1
4	−1	−1	−1.8	−6.7	−1.2	−1.7	−1.8	−15	L2
5	−0.9	−1	−1.9	−6	−1	−1.3	−1.9	−13.6	L2
6	−0.8	−1	−2.1	−5	−0.9	−1.3	−2.1	−12.5	L2
7	−1	−1.1	−2.8	−3.9	−0.9	−1.7	−3	−10.6	L2
8	−1.1	−1.4	−3.8	−2.9	−1	−2	−4.2	−8.1	L2
9	−1.2	−1.8	−5	−2.1	−1	−2.2	−6.2	−6	L2
10	−1.3	−2.1	−6.5	−2	−1.1	−2.7	−8.9	−4.2	L2
11	−1.5	−2.3	−8.3	−2.2	−1.3	−3	−12	−3.3	L2
12	−1.8	−2.9	−10.4	−2.7	−1.4	−3.3	−14.8	−3	L2
13	−2	−3.5	−13	−3.2	−1.5	−4.1	−16.1	−3.1	L2
14	−2.4	−4.1	−16.2	−3.9	−1.8	−5	−16	−3.2	L2
15	−2.8	−4.5	−18.9	−4	−2	−6	−14.5	−3.6	L2
16	−3.1	−4.9	−20	−5.1	−2.2	−7	−13	−4.1	L2
17	−3.6	−5.1	−20.1	−6	−2.5	−7.5	−12.5	−4.7	L2
18	−3.7	−5.2	−20.2	−6.1	−2.8	−7.5	−12.2	−4.9	L2

**Table 4:** Results of fitting the model curve (Gaussian) to the measurement results.

Resin	Absorber Content (%)	Thickness (mm)	Noise *B* (dB)	Amplitude *A* (dB)	Maximum Value *B + A* (dB)	Width *Δ* (GHz)	Max. Frequency *μ* (GHz)	Slope *A*/*δ* (dB/GHz)	*R*^2^ Value
L1	75	0.5	−4.21	−3.38	−7.59	2.27	9.10	1.49	0.970
L1	75	1	−4.30	−9.28	−13.58	3.24	9.51	2.86	0.988
L1	75	1.5	−4.01	−8.42	−12.44	2.94	8.88	2.87	0.996
L1	75	2	−3.62	−59.77	−63.39	11.18	−9.36	5.35	0.998
L1	80	0.5	−2.06	−3.99	−6.05	3.00	17.70	1.33	0.980
L1	80	1	−1.50	−8.50	−10.00	6.95	14.00	1.22	0.901
L1	80	1.5	−5.42	−7.58	−12.99	3.46	9.89	2.19	0.971
L1	80	2	−5.11	−9.56	−14.67	2.37	8.59	4.03	0.993
L2	75	0.5	−0.89	−2.71	−3.60	4.50	17.00	0.60	0.973
L2	75	1	−0.87	−4.03	−4.90	5.29	16.00	0.76	0.967
L2	75	1.5	−1.80	−18.20	−20.00	4.80	16.00	3.79	0.981
L2	75	2	−1.97	−4.68	−6.65	3.19	3.88	1.47	0.985
			−1.95	−4.19	−6.14	4.14	18.11	1.47	0.985
L2	80	0.5	−0.98	−1.52	−2.50	4.10	17.00	0.37	0.939
L2	80	1	−1.50	−6.00	−7.50	4.67	17.00	1.29	0.983
L2	80	1.5	−1.67	−14.70	−16.36	4.11	13.45	3.57	0.891
L2	80	2	−2.87	−13.90	−16.77	5.22	2.65	2.66	0.989
		2	−3.03	−1.87	−4.91	2.61	17.92	2.66	0.989

## Data Availability

The original data are stored at the Military Institute of Engineer Technology and are available upon request.
